# On the relationship between persistent delay activity, repetition enhancement and priming

**DOI:** 10.3389/fpsyg.2014.01590

**Published:** 2015-01-22

**Authors:** Elisa M. Tartaglia, Gianluigi Mongillo, Nicolas Brunel

**Affiliations:** ^1^Center for Neuroscience and Cognitive Systems @UniTn, Istituto Italiano di TecnologiaRovereto, Italy; ^2^Departments of Statistics and Neurobiology, University of ChicagoChicago, IL, USA; ^3^Centre de Neurophysique, Physiologie, Pathologie, Université Paris DescartesParis, France; ^4^Centre National de la Recherche Scientifique, Unités Mixtes de Recherche 8119Paris, France

**Keywords:** priming, neural network modeling, short-term memory, repetition enhancement, repetition suppression

## Abstract

Human efficiency in processing incoming stimuli (in terms of speed and/or accuracy) is typically enhanced by previous exposure to the same, or closely related stimuli—a phenomenon referred to as priming. In spite of the large body of knowledge accumulated in behavioral studies about the conditions conducive to priming, and its relationship with other forms of memory, the underlying neuronal correlates of priming are still under debate. The idea has repeatedly been advanced that a major neuronal mechanism supporting behaviorally-expressed priming is repetition suppression, a widespread reduction of spiking activity upon stimulus repetition which has been routinely exposed by single-unit recordings in non-human primates performing delayed-response, as well as passive fixation tasks. This proposal is mainly motivated by the observation that, in human fMRI studies, priming is associated to a significant reduction of the BOLD signal (widely interpreted as a proxy of the level of spiking activity) upon stimulus repetition. Here, we critically re-examine a large part of the electrophysiological literature on repetition suppression in non-human primates and find that repetition suppression is systematically accompanied by stimulus-selective delay period activity, together with repetition enhancement, an increase of spiking activity upon stimulus repetition in small neuronal populations. We argue that repetition enhancement constitutes a more viable candidate for a putative neuronal substrate of priming, and propose a minimal framework that links together, mechanistically and functionally, repetition suppression, stimulus-selective delay activity and repetition enhancement.

## 1. Introduction

In priming, stimuli are perceived/identified with greater accuracy and/or reduced reaction times if observers have been previously exposed to intact (i.e., repetition priming), noisy or related versions of them (i.e., semantic priming). Reaction times reductions can span from few milliseconds up to several dozen milliseconds, depending on different factors as, for example, the time between subsequent presentations (Vorberg et al., 2003). A single exposure to an item—the prime—has been shown to be sufficient for the behavioral facilitation to become apparent (Demb et al., 1995; Schacter et al., 1995; McMahon and Olson, 2007), although the effect gradually increases across multiple stimulus repetitions (Logan, 1990; Wiggs et al., 1997; Ostergaard, 1998). In visual object priming, slight across-repetitions modifications of the stimulus physical features, which do not significantly alter its appearance (e.g., small variations in orientation) can still yield priming although to a lesser extent than when identical stimuli are repeated (Ellis et al., 1989; Biederman and Cooper, 1991, 1992; Cooper et al., 1992; Srinivas, 1993, 1995). Priming occurs even if the exposure to the prime is not consciously recalled (Tulving et al., 1982), indicating that a contribution from explicit memory, which involves conscious recollection of past experiences, might be unnecessary. Accordingly, it has been suggested that priming is an *implicit* form of memory. Support to this view mainly comes from studies on amnesiac patients. It has been shown, at least in some cases, that priming of both familiar and novel objects is generally preserved in amnesiac patients, whose ability to explicitly recollect events is severely impaired (Musen and Squire, 1992; Squire, 1992; for a comprehensive review see Schacter and Buckner, 1998). Further hints on priming relying on separate mechanisms than those supporting explicit memory come from the observations that in elderly people, explicit recall and recognition are usually more strongly impeded than priming (La Voie and Light, 1994). However, the notion that priming does not involve explicit memory processes remains rather controversial (Ostergaard, 1999; Berry et al., 2010). Some forms of priming are extremely long lasting (and are likely reflecting long-term, experience-dependent changes; see, e.g., Schacter et al., 1993), others fade away after few seconds being overwritten by subsequent primes (Neely, 1991).

**Figure 1:**
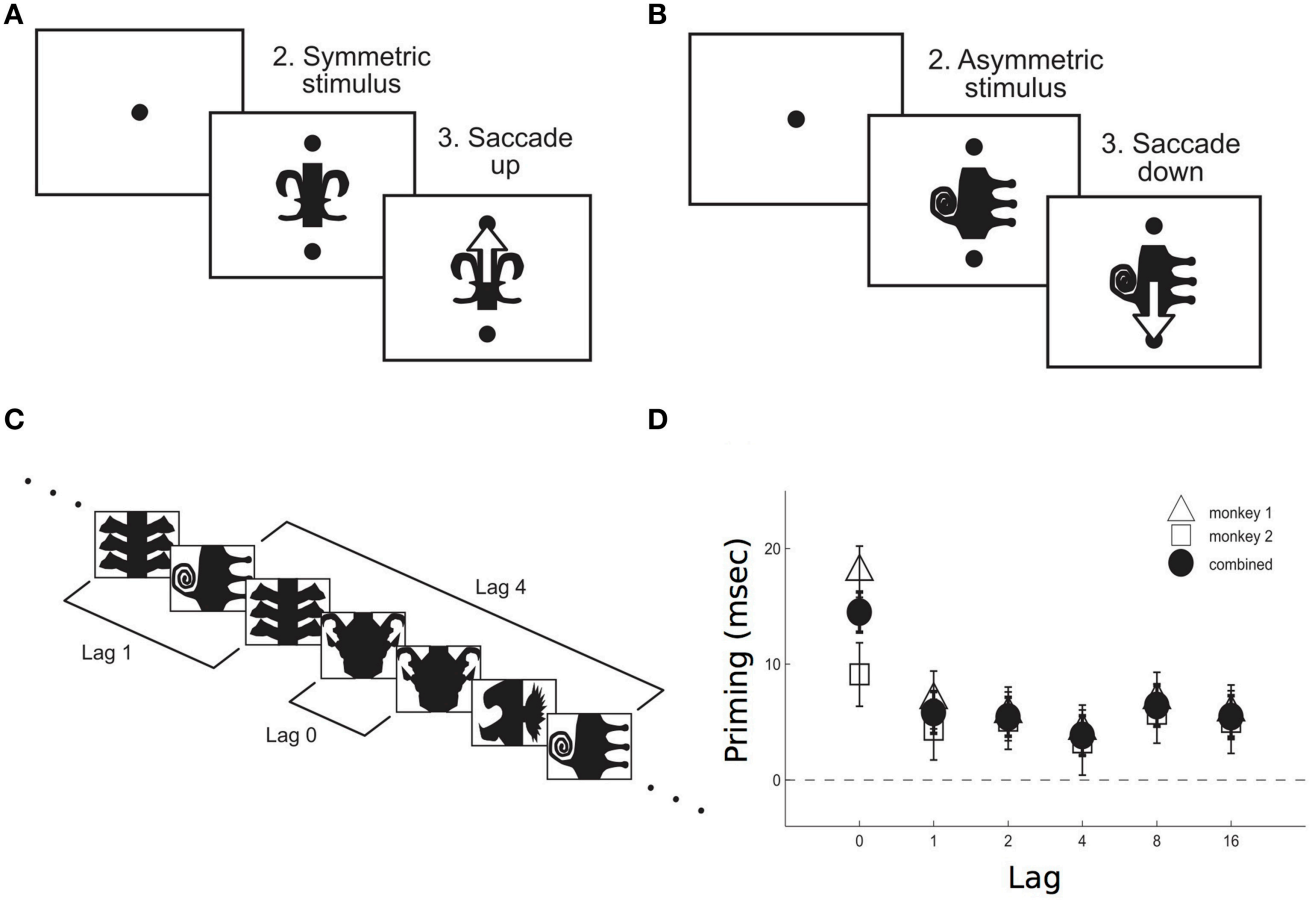
**Priming effect as measured in monkeys performing a symmetry decision task**. After a fixation period (200 ms), either a symmetric **(A)** or an asymmetric **(B)** stimulus appeared on the screen, together with two cues. Monkeys were required to make a saccade upwards/downwards in response to a symmetric/antisymmetric stimulus. Priming was measured for both stimuli repetitions across different lags **(C)**. Priming effect–as the difference between reaction times in response to the first and the second presentation of a stimulus- was larger when no other stimulus was interleaved between the two presentations, i.e., 0 lag **(D)**. Adapted from McMahon and Olson (2007).

Identifying the neural mechanisms which mediate priming has been difficult, on one hand, because of the difficulties to study it via neurophysiological recordings. Forms of priming which involve semantic judgments are difficult to assess in non-human primates; visual priming evaluation in monkeys is usually impeded by task overtraining, which causes behavioral performance to be already at ceiling at the time of the recordings, leaving hardly any room for further improvement [with the remarkable exception of the study of McMahon and Olson (2007); Figure 1]. On the other hand, priming studies on human observers via FMRI and ERP have provided rather controversial, in fact contradictory in some cases, results on the underlying dynamics of neural activity (e.g., Henson, 2003). Several theories concerning the putative neural mechanisms of priming have been put forward. One theory holds that the neural mechanism accountable for priming is *repetition suppression* (for reviews see Wiggs and Martin, 1998; Grill-Spector et al., 2006; Schacter et al., 2007; Gotts et al., 2012), that is the reduction in neural activity upon stimulus repetition^1^ Importantly, such a reduction in neural activity is not due to unspecific *fatigue* effects. A cell which shows suppression in response to a repeated stimulus can still strongly respond to a novel one, which rules out the possibility that reduced response is a consequence of activity-dependent adaptation at the single-neuron level. Activity-dependent depression at the synaptic level is ruled out by the following experimental observation. Response suppression is readily apparent with inter-stimulus intervals up to 5 s within a trial. On the other hand, it completely disappears after inter-trial intervals as short as 1–2 s (when the test stimulus of the previous trial is the same as the sample stimulus of the next one) (Miller et al., 1993). Indeed, priming and repetition suppression share, in some situations, several features (Wiggs and Martin, 1998). For example, priming can be induced by a single presentation of a visual stimulus. Likewise, repetition suppression has been observed after the very first repetition of a novel stimulus (Li et al., 1993; McMahon and Olson, 2007). Priming builds up over several stimulus presentations; similarly, neural activity in response to multiple repetitions of novel stimuli gets gradually suppressed as stimuli become familiar (Baylis and Rolls, 1987; Miller et al., 1991; Li et al., 1993; Xiang and Brown, 1998). Both priming and repetition suppression are thought to be automatic processes as they both appear to be dissociated from recollection and recognition performance (Miller and Desimone, 1994; Desimone, 1996). Consistently with this idea, repetition suppression is observed also during passive fixation tasks (Miller and Desimone, 1991; Riches et al., 1991; Miller et al., 1993; Vogels et al., 1995; Sawamura et al., 2006; Lehky and Sereno, 2007; Liu et al., 2009; Qi et al., 2012). Despite these common features, it remains unclear how a reduction in neural activity can yield a faster and more accurate behavioral performance. Several neural mechanisms have been proposed in the effort to reconcile priming with repetition suppression effects on neural activity (for reviews see Grill-Spector et al., 2006; Gotts et al., 2012).

Importantly, neurophysiological studies have shown that repetition suppression is, in many cases, accompanied by an–often smaller but significant- proportion of neurons whose response is enhanced upon stimulus repetition. Concurrently, a fraction of the recorded cells displays a selective activation in the delay period separating subsequent stimulus repetitions. In the context of neurophysiological recordings, repetition enhancement and persistent activity, involving a minority of the sampled neurons, have largely been neglected as plausible neural correlates of priming, while they are widely considered as implicated in short-term memory tasks (e.g., Desimone, 1996).

Here, we first review neurophysiological studies which have consistently exposed *both* enhancement and suppression of neural activity *upon* stimulus repetition, as well as persistent delay activity *between* stimulus repetitions. These patterns of neural activity have been observed in the context of behavioral tasks involving or not an explicit recognition memory component. Secondly, we propose a theoretical framework which functionally links persistent delay activity and suppression/enhancement effects and provide a mechanistic explanation of priming. In our framework, although repetition suppression involves the majority of neurons, repetition enhancement of activity in a small fraction of cells–brought about by persistent delay activity- is responsible for the faster behavioral performance observed in priming.

## 2. Evidence of repetition enhancement and persistent delayed activity in neurophysiological recordings

### 2.1. Neural recordings in DMS tasks

A substantial amount of data on the neural correlates of repeated stimulus presentation has been collected via neurophysiological recordings^2^ in the context of recognition memory tasks, as, for example, a *delayed match to sample* (DMS) task, which requires a comparison between two stimuli–a sample and a test- separated by a temporal delay and, thereby, an explicit retrieval of previously seen stimuli. A reduction of activity (*repetition suppression* effect) has been observed in a significant fraction of the recorded neurons, in several cortical areas and with various visual stimuli, upon repetition of the sample (i.e., when the test matches the sample; for reviews see Ringo, 1996; Grill-Spector et al., 2006). However, in a typically smaller fraction of cells the opposite trend has been reported, with neural responses significantly increasing upon stimulus repetition (*repetition enhancement* effect). Many of these studies also report a persistent neural discharge in the delay period separating consecutive stimulus presentations–i.e., *persistent delay activity*.

In a DMS task with simple geometric shapes, when comparing neuronal responses in inferotemporal cortex to the first (sample) and the repeated (match) stimulus presentation, Baylis and Rolls (1987) found that, while 19% of the visually responsive neurons had a stronger activation to the sample, about 5% of them responded more strongly to the match stimulus (for the visually selective neurons, these percentages were 19% and 7%, respectively). A slightly smaller percentage of neurons (about 6%) persistently fired during the delay period separating the two stimulus presentations (Baylis and Rolls, 1987). With the same kind of task and stimuli (drawn from a set of objects with different shape, color, and size), Riches et al. (1991) reported that 34% of the differentially responsive neurons in inferotemporal and rhinal cortex (71% in the hippocampal formation and parahippocampal gyrus) responded more vigorously to the second than to the first stimulus presentation. Between 20% and 30% of the visually responsive neurons, showed persistent delayed activity between the two subsequent presentations, with no significant difference between the various cortical areas (Riches et al., 1991). Using complex colored pictures of objects or textures and patterns in a DMS task, Miller et al. (1993) found that about 10% of the neuronal responses in inferotemporal cortex to match stimuli were enhanced with respect to the sample (slightly more than 20% if one considers only differentially responsive cells), with the remaining fraction showing the opposite behavior. About a quarter of the recorded cells showed sample selective persistent activity in the delay period. However, this activity did not survive the presentation of intervening stimuli between sample and match (Miller et al., 1993). In an alternative form of the DMS task in which the animal was required a motor response only when the test stimulus matched the sample, disregarding intervening stimulus repetitions (the “ABBA” task), the percentage of inferotemporal cortex cells showing an enhanced response to the final sample repetition increased to almost 20% (35% of the differentially responsive cells) (Miller and Desimone, 1994). Again, persistent delayed activity was observed in many cells but was disrupted by the presentation of a distractor.

Interestingly, both the percentage of neurons showing repetition enhancement and the percentage of those exhibiting sample selective persistent delay activity increased in prefrontal cortex, reaching, respectively, 42% and 33% of the responsive cells; response enhancement upon stimulus repetition was not only more common in prefrontal than in inferotemporal cortex, it was also greater in magnitude [Miller et al., 1996; similar percentages have been observed by Rainer et al., 1999].

Variable proportions of (differentially responsive) cells showing repetition enhancement (from 5% to 60%), as well as persistent activity, have been reported in posterior parietal cortex in a spatial version of a DMS task, in which monkeys were required to judge whether the locations of two sequentially presented stimuli were the same or different (Steinmetz et al., 1994; Constantinidis and Steinmetz, 2001; Rawley and Constantinidis, 2011).

Lui and Pasternack (2011) found almost 20% among the 171 recorded cells in area MT showing stronger responses to the repetition of a previously presented stimulus in a DMS task with motion stimuli. Importantly, these cells were shown to be the most robust predictors of monkeys' choice. Persistent activity selective for the identity of the sample direction was found early in the delay, and only rarely such activity persisted throughout the entire length of the delay (Lui and Pasternack, 2011).

In a DMS task with distractors, Hayden and Gallant (2013) found that almost half of all recorded cells in V4 showed sample selective delay activity which persisted also during the presentation of the distractors. More than 30% of all recorded neurons showed an enhanced response at match presentation compared to the response to the same stimulus presented as a distractor. Only 10% of neurons showed a suppression effect. Notably, 25% of all sampled cells showed both selective persistent delay activity and match enhancement (Hayden and Gallant, 2013).

Notably, evidence of both an early enhancement and a later suppression of the average activity of a population of cells in inferotemporal cortex have been found upon stimulus repetition (Woloszyn and Sheinberg, 2009). The authors report that, in a DMS task with complex objects pictures, only the repetition of the cells' preferred stimulus elicited the early enhanced response; such signal, they suggest, could be the most relevant to the monkey for the execution of the behavioral task. Again, selective persistent delay activity was found, which survived a distractor presentation.

### 2.2. Neural recordings in other delayed response tasks

Electrophysiological recordings on monkeys performing a DMS task allow to assess neural activity in response to repeated stimuli (i.e., when the sample stimulus serves as a *prime* for the subsequent match), thereby providing a measure of the neural correlates of repetition priming. Recordings on monkeys performing a paired associate task (e.g., Sakai and Miyashita, 1991; Naya et al., 1996), which is often used to probe the effects of associative memory on neural activity, have been suggested to provide valuable information on the neural mechanisms underlying semantic, rather than repetition priming (Brunel and Lavigne, 2009).

In semantic priming, reaction times in response to a test stimulus are reduced if the test is preceded by a conceptually or semantically related prime. In a paired associate task, the prime and the subsequent test consist in a pair of visual stimuli that the monkey has previously learned to associate. In each trial the monkey has to recognize whether the prime is followed by its pair-associate or by a different test stimulus. Although facilitatory effects on the behavioral performance are hard to observe on overtrained animals, priming-like effects on reaction times related to neurons' spike rates have been observed when the prime is followed by its pair-associate test (Erickson and Desimone, 1999). It is plausible, then, that the neural activity recorded in such condition might well-reflect the dynamics underlying priming of semantically and/or conceptually related stimuli.

Neurophysiological recordings in the prefrontal cortex of monkeys performing a paired associate task–with pictures of real world objects- have exposed, in analogy with the DMS task, both enhancement and suppression of neural activity in response to the test stimulus following its pair-associate prime–when compared to the response to the same test stimulus following a neutral prime. Interestingly, in contrast with the DMS task, the proportion of neurons showing enhanced activity to the paired associate test was found to be significantly greater than the proportion showing suppression. Concurrently, out of 181 responsive cells, 87 showed selective persistent activity during the delay separating the prime and the test (Rainer et al., 1999).

In a delayed match to category task, in which monkeys have to recognize whether the test matches the category of the previously presented sample–i.e., the related prime-, Freedman et al. (2003) found that 49% of the differentially responsive cells found in prefrontal cortex showed higher activity to matches than non-matches–where a non-match consists in the same test stimulus but following a neutral prime, which belongs to a different category- and 51% showed the opposite effect. The proportion of cells showing match enhancement was even higher in inferotemporal cortex where 63% of neurons showed more activity to matches while the remaining showed more activity to non-matches. Persistent delay activity selective to the sample category was found in 9% of all recorded inferotemporal neurons and 18% of the recorded prefrontal neurons (Freedman et al., 2003).

### 2.3. Neural recordings in passive fixation tasks

Repetition enhancement as well as persistent delayed activity, along with repetition suppression, have also been found during passive fixation, a task that does not involve a recognition component and, therefore, does not require active maintenance of previously seen stimuli.

**Figure 2:**
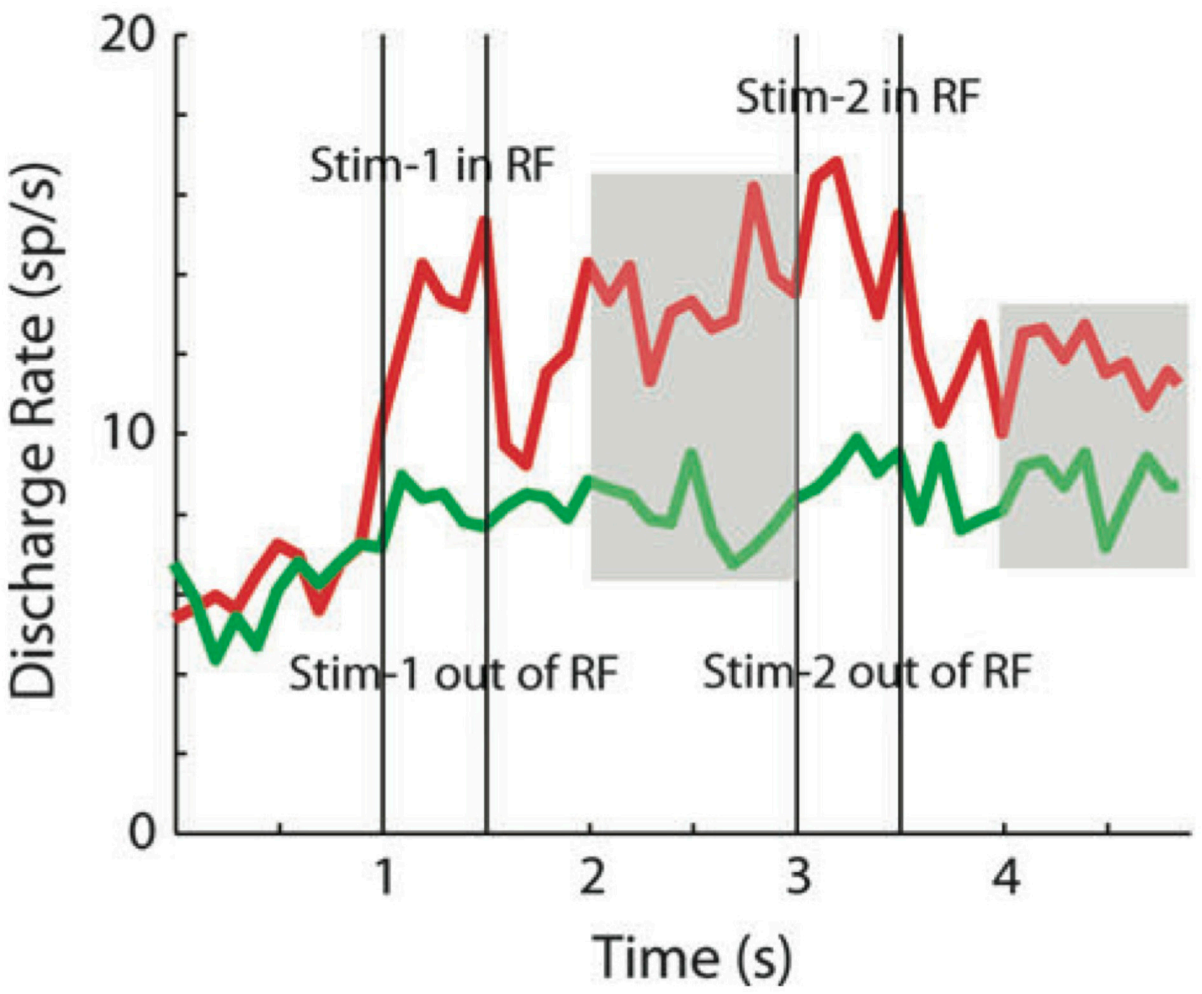
**Repetition enhancement and persistent delay activity**. Average population activity (29 cells in prefrontal cortex) in response to the repetition of a stimulus inside (red curve) and outside (green curve) the neuron's receptive field. Vertical bars indicate the time of the first (sample) and the second stimulus presentation (match). The activity between sample and match as well as after the match offset, is significantly higher than baseline (persistent delay activity–indicated by the gray areas-). Although the authors do not analyse neural activity during match presentation, the repetition enhancement effect is apparent when both stimuli were presented inside the receptive field. Adapted from Meyer et al. (2007).

Qi et al. (2012) recorded neuronal activity from the lateral prefrontal cortex of monkeys after they were trained to perform a spatial DMS task and compared these responses to those obtained from the same animals, before learning the task, when the identical stimuli were presented for passive viewing. Repetition enhancement and persistent activity were apparent even before training. Changes in both effects observed after training were quantitative rather than qualitative, involving a higher percentage of neurons with a higher mean firing rate (Qi et al., 2012). In a previous study from the same group, the authors had already reported a small fraction of cells in prefrontal cortex firing persistently during the delay period separating the repetition of a stimulus in a passive fixation task. Interestingly, these cells also exhibited an apparent enhanced response to the second stimulus presentation (Figure 2), although the effect has not been statistically quantified (Meyer et al., 2007).

Vogels et al. (1995) compared neural responses to stimulus repetitions in inferotemporal cortex in a passive fixation task and in a highly trained DMS task. While the passive fixation task involved the repetition of novel stimuli, in the DMS task the stimuli were highly familiar. Out of the 49 selected cells, which responded equally to both novel and familiar stimuli, most exhibited an overall repetition suppression effect in both tasks. However, six percent of neurons showed repetition enhancement in the DMS task. The proportion of cell showing repetition enhancement in the fixation task did not reach significance, likely because of the small sample of recorded cells. Seven percent of the cells recorded in the DMS task showed persistent delayed activity, which was strongly reduced in the fixation task (Vogels et al., 1995).

In remarkable resemblance with priming (Ellis et al., 1989; Biederman and Cooper, 1991, 1992), both repetition suppression and repetition enhancement have been shown to survive object transformations of size and location upon repetition in inferotemporal cortex (Lueschow et al., 1994).

Table 1 recapitulates all the reported data. In the effort to facilitate the comparisons across studies, the percentages of suppression and enhancement reported refer to the subset of differentially responsive cells, while the percentage of cells showing persistent activity refers to the whole recorded sample.

**Table 1:** **First column: cortical region of recordings. Second column: task; oDMS = object DMS; sDMS = spatial DMS; DDD = delay direction discrimination; DPA = delay paired associate; DMC = delay match to category; PF = passive fixation. Third column: percentage of differentially responsive neurons showing suppression. Fourth column: percentage of differentially responsive neurons showing enhancement. Fifth column: percentage of all recorded neurons showing persistent activity**.

**Region**	**Task**	**%MS**	**%ME**	**%PA**	**References**
ITC	oDMS	81%	19%	6%	Baylis and Rolls, 1987
HF + PHG	oDMS	29%	71%	20%	Riches et al., 1991
TE + RH	oDMS	66%	34%	20%	Riches et al., 1991
ITC	oDMS	77%	23%	25%	Miller et al., 1993
ITC	oDMS	65%	35%	many	Miller and Desimone, 1994
ITC	oDMS	72%	28%	19%	Miller et al., 1996
ITC	oDMS	89%	11%	7%	Vogels et al., 1995
PFC	oDMS	40%	60%	33%	Miller et al., 1996
PPC	sDMS	40%	60%	few	Rawley and Constantinidis, 2011
PPC	sDMS	92%	8%	ND	Steinmetz et al., 1994
PPC	sDMS	95%	5%	ND	Constantinidis and Steinmetz, 2001
MT	DDD	66%	34%	54% early delay	Lui and Pasternack, 2011
V4	oDMS	23%	77%	49%	Hayden and Gallant, 2013
PFC	DPA	42%	58%	48%	Rainer et al., 1999
PFC	DMC	51%	49%	18%	Freedman et al., 2003
ITC	DMC	37%	63%	9%	Freedman et al., 2003
PFC	PF	68%	32%	NR	Qi et al., 2012
PFC	sDMS	50%	50%	NR	Qi et al., 2012
PFC	sDMS	73%	27%	ND	Lueschow et al., 1994

*ND = persistent activity cannot be observed since other stimuli are presented during the delay; NR = percentages are not reported although persistent activity is observed*.

All above-mentioned studies involve repetition of highly familiar stimuli in the context of highly trained tasks, involving or not a voluntary memory component. It has been suggested that stimulus familiarity could be related to the amount of repetition enhancement–i.e., only the repetition of familiar stimuli can elicit increased neural activation (Rugg et al., 1995). However, enhancement has been found upon repetition of novel stimuli in both a highly trained recognition memory task (Baylis and Rolls, 1987; Rolls et al., 1989; Xiang and Brown, 2004) and a passive fixation task (Tovee et al., 1996). In this latter study, neural activity was recorded from 21 face selective neurons in ITC while the monkey was passively exposed to a series of novel ambiguous and unambiguous face stimuli. In one third of the cells an enhancement of neural response to repeated ambiguous stimuli was observed after repeated exposure (for a total of 5 s) to its unambiguous version. Note that, although the cells sample is quite limited, the authors report a probable underestimation of the enhanced population since the recorded cells were not specifically tuned to the unambiguous face.

Several studies have pointed out that the duration of both repetition suppression and enhancement effects might depend on the stimulus familiarity (Miller et al., 1993; Ringo, 1996; Ranganath and Rainer, 2003; Sawamura et al., 2006; Liu et al., 2009). Repetition of familiar stimuli elicits transient suppression and/or enhancement of neural responses, occurring over short time scales–it has been estimated that neurons recover their responsiveness after few seconds (Miller and Desimone, 1991; Liu et al., 2009). To the contrary, repetition effects on neural activity measured with novel stimuli can be quite persistent, occurring over long time scales–from hours (Fahy et al., 1993; Li et al., 1993; Kobatake et al., 1998; Xiang and Brown, 1998), to several days (van Turennout et al., 2000). Such long-term effects usually require a substantial amount of repeated exposures (but see Li et al., 1993) and likely involve long-term synaptic plasticity processes through which stimuli become familiar. Such types of neural changes might underlie extremely long lasting forms of priming (Cave, 1997; Mitchell, 2006), which are more likely related to experience-dependent behavioral changes like those observed in perceptual learning (see e.g., Ahissar and Hochstein, 2004). The mechanisms underlying these long-lasting forms of priming are beyond the scope of this review and will not be discussed further.

## 3. Mechanistic models of priming

What are the mechanisms of repetition priming? What provokes the faster response to the repetition of a stimulus, compared to its first presentation? The basic tenet of all models that have been proposed is that network(s) underlying priming have a different response to the second stimulus than to the first, and that this difference in responses lead to the observed differences in behavior. A major constraint on models is the observation, in the majority of cells, of *repetition suppression*, while in a smaller but significant fraction of cells, *repetition enhancement* is observed. Different scenarios compatible with these constraints have been outlined by Gotts et al. (2012): (1) an increase in firing rates in response to the second stimulus could occur faster than the response to the first (*facilitation*); (2) while the majority of cells would decrease their firing rates, a small subpopulation of cells–most responsive to the stimulus- retain their level of activation upon repetition (*sharpening*); (3) the *synchronization* between neurons could increase from the first presentation to the second, thereby leading to a faster behavioral readout in spite of a decrease in mean rates.

The next question that needs to be answered is then what are the mechanisms leading to such changes? Conceptually, one could imagine at least three types of mechanisms for why the response to a repeated stimulus might be different to the response to the first: (i) a single neuron mechanism: the first stimulus could trigger an intrinsic ionic current, that would change the state of the neuron, therefore modifying its response to subsequent stimuli; (ii) a synaptic plasticity mechanism: the first stimulus could trigger changes in the efficacies of synapses, which would then lead to changes in the response to subsequent stimuli; (iii) a network mechanism: the first stimulus could switch the network to an activity state that differs from the state in which the network was before the stimulus, which would then modify the response of the network to subsequent stimuli. In the following, we will focus on mechanisms (ii) and (iii). The first scenario has, to our knowledge, not been explored by modeling studies.

### 3.1. Models relying on synaptic plasticity mechanisms

Synaptic plasticity refers to a broad range of phenomena that describe dynamics of synaptic efficacy over time scales of hundreds of seconds (short-term plasticity) to much longer time scales (long-term plasticity).

Gotts (2003)–see also Gotts et al., 2012- proposed that short-term synaptic depression leads to a firing rates decrease upon stimulus repetition, but also to a more synchronous regime of activity, i.e., spiking activity will become more correlated both within and across cortical regions. Because of the increase in synchrony, a downstream cell reaches its firing threshold faster, yielding the reduction of reaction times observed in behavioral priming (in spite of reduced firing rates).

The model is able to reproduce the so-called *scaling effect* of neural responses occasionally observed upon stimulus repetition of novel stimuli, according to which the most responsive neurons exhibit the largest suppression, in stark contrast with the sharpening model (Li et al., 1993; McMahon and Olson, 2007). However, Weiner and Grill-Spector (2012) pointed out that increased synchrony within *and* across cortical areas predicted by the model appears to be in disagreement with LFP data, which rather indicate “an anticorrelated relationship between local and inter-areal synchrony as a function of repetition.” Furthermore, both a reduction and an increase of synchrony among neurons have been observed experimentally upon stimulus repetition (e.g., Gruber and Muller, 2002, 2005; Brunet et al., 2014).

Models implementing long-term synaptic plasticity mechanisms assume that long-term synaptic changes (i.e., long-term potentiation and depression) induced by a single presentation of a stimulus are strong enough to induce observable changes in the response of the network to a subsequent presentation of the stimulus. Intuitively, Hebbian-like synaptic changes typically tend to favor the stability of the network state that was driven by the stimulus; this then leads to a faster response to a second presentation of the stimulus (Becker et al., 1997).

In a recurrent network model originally designed to explain the improvement of performance observed in a delayed-match-to-multiple-sample task with novel vs. familiar stimuli, a single stimulus repetition, via a *one-shot*, Hebbian-like change of the synaptic efficacies, induces a population response which is higher—on average—than that elicited upon the first presentation (Yakovlev et al., 2008). Again here, the enhancement of neural activity potentially leading to priming effects is induced via stimulus induced synaptic plasticity.

Moldakarimov et al. (2010b) implemented such a Hebbian scenario in a two-layered neural network model. In this model, a stimulus impinging on the network induces a distribution of activity on layer one neurons. Hebbian connections strengthen synapses between active neurons, while synapses to weakly active neurons are depressed. The more a stimulus is repeated, the more initially weakly responsive neurons are silenced, leading to a sparser representation. The sharpening of the stimulus representation in the early visual areas leads to more selective activation of representations in the higher visual areas, i.e., in the upper layer of the model consisting of a winner take all network. Upon stimulus repetition, the differential activation in layer one sustained by hebbian connections, facilitates the competition between populations in layer two, thereby the winning unit suppresses the other units and reaches the threshold faster, shortening the reaction time of the network. In the Moldakarimov et al. (2010b) model, suppressed neural activity upon stimulus repetition results in *sharpening* in early visual areas, which, in turns, yields a facilitation in reaching a decision–i.e., a shorter response latency- in higher cortical areas. Note that this model reproduces both *repetition suppression* effects (in neurons of the first layer that are only weakly activated by the stimulus) and *repetition enhancement* (in the “winner” neurons of the second layer).

In a spiking neurons model version of their previous work, Moldakarimov et al. (2010a) could reproduce the reduction of gamma oscillations observed in priming combining sharpening with a reduction of synchrony among excitatory neurons.

### 3.2. Models relying on changes in network activity state

A different class of models relies on attractor dynamics. Hebbian, long-term synaptic modifications induced by external stimuli can lead to the creation of selective attractor states, in which a subpopulation of neurons maintain an elevated, persistent activity following the presentation of a particular stimulus (Amit, 1995; Wang, 2001; Brunel et al., 2004). In this scenario, the fact that the network goes to a selective attractor state following presentation of the stimulus means that when the stimulus is shown for the second time, the network state is different from the one when the stimulus was shown for the first time. In particular, specific populations of neurons could be initially more active, which would lead again to a faster response upon stimulus repetition.

This idea was investigated by Brunel and Lavigne (2009), which showed that semantic priming effects can be quantitatively reproduced in an attractor neural network framework, in which separated neural populations code, respectively, for the test stimulus, its semantically associated primes and neutral primes (Brunel and Lavigne, 2009). Because of the structure of the synaptic connectivity, the presentation of a semantically associated prime induces activation also in the population coding for the test stimulus, causing its average firing rate to increase throughout the delay period (Mongillo et al., 2003). Hence, at test stimulus onset, the activation level of the corresponding neural population is higher than baseline (i.e., than its average firing rate in the absence of any stimulus presentation), leading to an enhanced and faster response to the upcoming test stimulus. On the other hand, the presentation of a neutral prime, does not induce persistent delay activity in the population coding for the test which, therefore, displays a slower and weaker response upon test presentation. Consequently, a read-out neuron downstream reaches its target *decision* threshold faster when the prime is associated to the test—rather than when a neutral prime precedes it—eventually leading to the shorter reaction times behaviorally observed.

The model is able to reproduce the dynamics of priming effects and, in particular, the dependency of reaction time reductions on the duration of the delay period separating prime and test presentation -or stimulus onset asynchrony (SOA)- observed in the context of semantic priming. Moreover, the model is consistent with neurophysiological recordings of monkeys performing a paired-associate task, in which a prime is followed by its paired-associated test–previously learned- or by a neutral test stimulus. The model relies on persistent delay activity, which leads to enhancement of the neural response upon associated test presentation, but leaves unaddressed the issue of neural activity suppression widely observed in the context of priming effects.

Finally, we outline a model of repetition priming that reconciles the three neural correlates consistently observed upon stimulus repetition—repetition suppression, repetition enhancement and persistent delay activity (Tartaglia et al., in press). In this framework, the enhancement of neural activity involves a small fraction of cells, in agreement with neurophysiological observations, but is to be held accountable for the priming effect.

In our model, implemented via a standard attractor neural network of excitatory and inhibitory spiking neurons, both repetition suppression and repetition enhancement arise from the dynamical interplay of the broad selectivity of visual responses–consistently with several observations that cortical neurons typically show visual responses to a large fraction of stimuli, even after such stimuli have become fairly familiar (Woloszyn and Sheinberg, 2012; Wohrer et al., 2013)–and persistent delayed activity.

**Figure 3:**
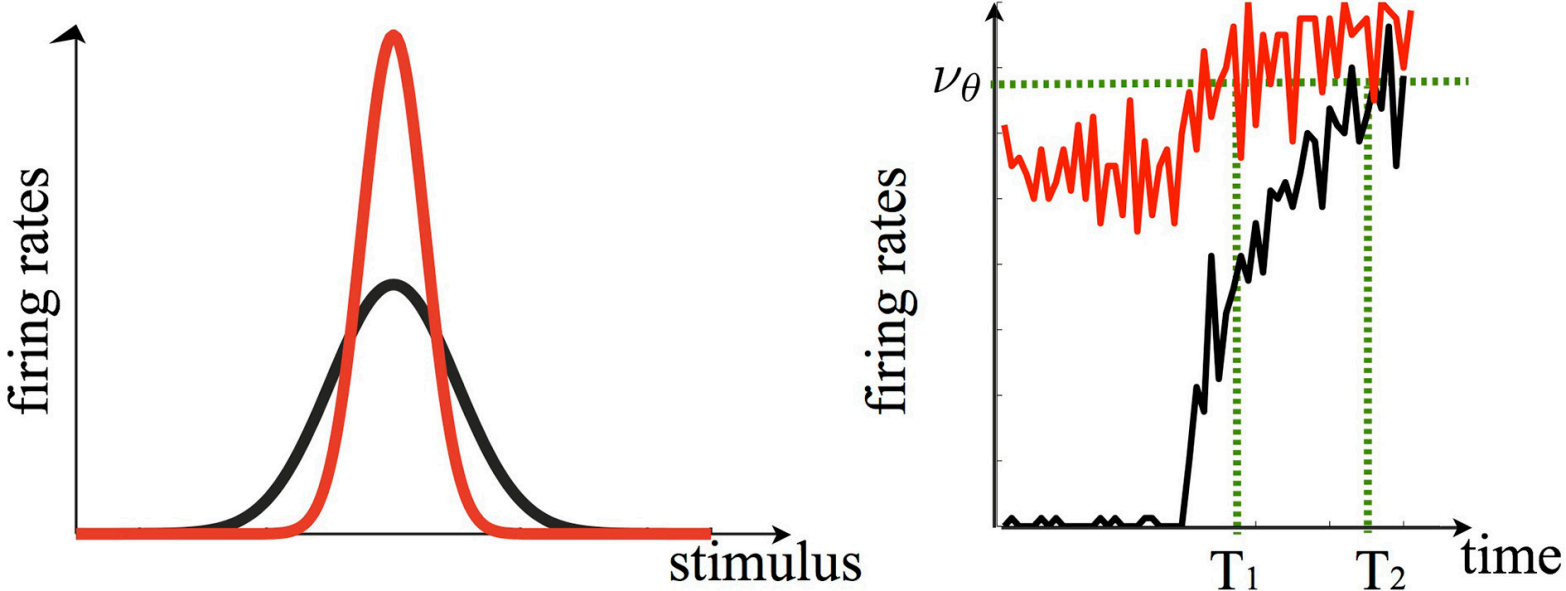
**In our model persistent delay activity is instrumental to both the suppression of activity of cells less selective to the sample and the enhancement of activity of those most selective to it**. In this framework, the tuning curve of a given cell upon stimulus repetition is sharpened (red curve, **left panel**). Note that in the original sharpening model the response to the cell's preferred stimulus would not change upon repetition, i.e., the peaks of the red and black curve would overlap. The *enhanced* sharpening stems from persistent delay activity. The **right panel** shows how, following the sample offset, the population of cells which are mostly selective to it continues to fire persistently during the delay period (red curve to the left of the black dashed line, which indicates stimulus onset). Upon sample repetition, the population activity, starting off from a higher level with respect to the first presentation (black curve), ramps up faster–yielding the repetition enhancement effect- eventually reaching a recognition threshold (ν_θ_) faster (*T*_1_ < T_2_). Curves are generated via the spiking neuron simulation in Tartaglia et al., (in press).

Following the first stimulus presentation (sample/prime), the population of neurons which responds the most to it—a small fraction of the whole network- keeps firing persistently, serving as the memory trace of the stimulus. Such persistent activity, in turns causes, by increasing the overall network inhibition, a suppression of activity in the remaining populations, which constitutes the majority of neurons. Upon stimulus repetition (match/target), the changes in the patterns of neural activity brought about by the persistent activity, induce an enhanced response in the most selective neurons and a suppressed response in the less selective ones. Hence, as in the original sharpening model, the responses of the weakest neurons decrease when a stimulus is repeated. However, the concurrent response enhancement of a small fraction of neurons now carries the critical information about the stimulus and can yield, via a suitable read-out mechanism, the more accurate/faster behavioral performance observed in priming (Figure 3). The model is also robust to distractors presentation, i.e., both suppression and enhancement response to the match/target stimulus survive the presentation of interleaving stimuli. Both effects decrease as the number of distractors decrease, consistently with neurophysiological data (Miller et al., 1993)

Our model essentially relies on persistent delay activity whereby neurons belonging to the memory representation–i.e., those that fire persistently during the stimulus retention period- respond more promptly upon stimulus repetition–as in Brunel and Lavigne (2009). Such faster response is transmitted to a read-out neuron downstream which reaches the threshold for recognition faster eventually leading to priming (Figure 3). Since the activity of the majority of neurons outside the memory representations is suppressed, the net effect upon stimulus repetition, as in Moldakarimov et al. (2010a), is an increased sparseness of the neural representation, in accordance with the sharpening model, with fewer neurons belonging to the memory representation whose activity is enhanced. Such activity pattern can be easily reversed upon the very first repetition of a new stimulus: a cell whose activity is strongly suppressed following the repetition of a given stimulus A, can show enhancement in the subsequent trials upon repetition of its preferred stimulus B.

In our model, repetition enhancement targets preferentially the cells which exhibit the strongest response to the sample, consistently with some of the data reviewed above (Woloszyn and Sheinberg, 2009), and which display selective sustained activity during the delay period^3^. Accordingly, repetition enhancement would be difficult to observe in neurophysiological recordings in which the sampling procedure was not optimized to find the best stimulus which drives the cell during the delay period. Likewise, the proportional scaling effect observed by some authors (Li et al., 1993; McMahon and Olson, 2007) might be a byproduct of the chosen sample selection procedure. Li et al. (1993) did not actively look for the cells' preferred stimuli during the delay, consequently, the stimulus which elicited the highest visual response, and the highest repetition suppression effect, was not necessarily active at enhanced rates during the delay period.

Such an explanation would be also consistent with the fact that no repetition enhancement has been observed in those studies which reported proportional scaling (Li et al., 1993; McMahon and Olson, 2007). Finally, again in contrast to proportional scaling, the increase in sparseness (due to repetition suppression) accompanied by an increased firing rate to the best stimulus (due to repetition enhancement), are well in agreement with the neural changes observed upon multiple presentation of novel stimuli (Rainer and Miller, 2000; Woloszyn and Sheinberg, 2012). However, the long lasting repetition effects on neural activity observed in these studies likely require long-term synaptic mechanisms which gradually increases neuron's selectivity as the stimuli get familiar. In our model, enhancement and suppression result from modifications of the transient network response following stimulus presentation and as such have a relatively brief life-span, persisting for periods of the order of 1 s of stimulus presentation, well within the neurophysiological probed intervals.

One of the essential constituents of our model is persistent delayed activity, widely considered to be a major neuronal correlate of temporary memory storage. Several studies reviewed above have shown that persistent activity, as well as repetition suppression and repetition enhancement, are concurrently observed in several areas, not only during short-term memory tasks, but also during repeated passive exposure, which does not require any active maintenance of previously seen stimuli. These observations support the intriguing assumption that, upon stimulus presentation, some form of memory trace is automatically activated, regardless of task demands. Such memory trace, encoded in the sustained patterns of neural activity, can then support different mnemonic processes such as recognition in DMS tasks, associative recall in paired-associate tasks, or simply faster stimulus processing in priming, by modifying the tuning properties of the underlying neural representations. In these terms, our mechanistic interpretation is consistent with the so called “abstractionist” theories according to which priming stems from the reactivation of (voluntary or involuntary) preexisting memory representations (Henson, 2003; Turk-Browne et al., 2006), although priming for visual shapes has been shown to occur even in the absence of such representations (Kersteen-Tucker, 1991).

## 4. Conclusions

Suppression of neural activity upon repetition of a prime has been thought to be a neural correlate of priming. Even though suppression often involves the majority of the recorded neurons, a fraction of the sampled population responds more vigorously to the prime repetition–i.e., repetition enhancement effect. Interestingly, in almost all the instances in which repetition suppression and repetition enhancement are observed, also persistent delay activity is observed. These patterns of neural activity seem to co-occur in several brain areas and in the context of tasks that implicate or not an explicit mnemonic component.

Here, we propose a new perspective according to which repetition enhancement and persistent activity, although involving a small fraction of neurons–which is nonetheless quantitatively consistent with the fraction of selective neurons typically observed in neurophysiological studies- have a pivotal role in the neural machinery underlying priming effects. In this framework, the modulatory effects of neural activity upon stimulus repetition are conditional to persistent delay activity, which, disregarding whether it represents active or passive temporary storage of the prime—is instrumental to priming effects.

In our account, the presentation of a prime entails changes in the *network state* which eventually lead to a faster stimulus processing upon repetition. In other models (Becker et al., 1997; Yakovlev et al., 2008; Moldakarimov et al., 2010b), priming is the result of stimulus induced changes in the *synaptic state* of the network.

Our scenario gives rise to intriguing experimental predictions. Any manipulation (e.g., pharmacological) weakening or destroying persistent activity should have a significant impact on priming, severely diminishing its amplitude or completely hindering it. Support to this idea comes from the observation that both priming and working memory are impaired by pharmacological interventions which enhance the GABA-ergic neurotransmitter system, e.g., via benzodiazepines. It has been shown that benzodiazepines hinder repetition suppression (Thiel et al., 2001) and attenuate priming (Thiel et al., 2001; Boucart et al., 2002), in some cases completely impairing it (Vidailhet et al., 1999). Likewise, benzodiazepines yield an overall slowing of working memory processes in humans (Mintzer and Griffiths, 2007), monkeys (Dean et al., 1982), and rats (Ohno et al., 1992; Cole and Hillmann, 1994). Moreover, dopamine has been shown to constraint persistent delay activity during the execution of a working memory task (Williams and Goldman-Rakic, 1995) and to have a detrimental effect on priming (Pederzolli et al., 2008).

### Conflict of interest statement

The authors declare that the research was conducted in the absence of any commercial or financial relationships that could be construed as a potential conflict of interest.

## Footnotes

^1^FMRI stimulus repetition paradigms have consistently reported reduced latency of the BOLD response peak as well as a decrease in its magnitude (on much longer time scales than those of reaction time reductions typical of priming.). It has been suggested that changes in the BOLD response upon stimulus repetition could be interpreted as a net shorter duration of neural activity, which would be the signature of the faster stimulus processing underlying priming effects (Henson, 2003). Here, we focus on repetition suppression as measured in single cell recordings. We invite the reader to refer to Grill-Spector et al. (2006) for a comprehensive review on repetition effects measured with fMRI and EEG/MEG.

^2^Evidence of repetition enhancement have been found also via ERP and fMRI (Schacter et al., 1995; Dolan et al., 1997; Gauthier, 2000; Henson et al., 2000; Fiebach et al., 2005; James and Gauthier, 2006), but we are not discussing them here.

^3^Note, however, that increasing the network heterogeneity level e.g., by increasing the sparseness of the synaptic connections, might yield response enhancement also on cells which neither preferentially respond to the sample nor exhibit persistent delay activity, but, by chance, receive a stronger recurrent input upon stimulus repetition.
